# Annotations

**Published:** 1900-09-01

**Authors:** 


### Annotations.

### Municipal Mother's Milk.

The experiment of the St. Helen's Corporation in supplying to the poor of the town sterilised humanised milk for infants has attracted considerable attention. As the departure was only inaugurated in August, 1899, it is a little premature to judge results at the present time, but some interesting facts in connection with the work are mentioned in the recently issued annual report of the Medical Officer of Health. The charge made for the milk is twopence per day's supply, and is payable in advance. Within three weeks of starting the municipal depôt was feeding 80 children daily, and by the end of the year there were 120 children on the books. Although commenced so late in the season the effect on the infant mortality and diarrhœa rate has been marked. The infant mortality rate for St. Helen's in 1899 was 157 per 1,000 births, against a mean rate of 172 in the preceding 10 years. Only on one previous occasion has the rate been so low, and never before has it been lower than the rate for England and Wales. This represents a saving of 60 infant lives. It is noteworthy, too, that in a year when the climatic conditions were so favourable to the prevalence of epidemic diarrhœa the death-rate from the disease was lower than during the past two years. Another novel departure made by the Health Committee is the appointment of a female sanitary inspector, part of whose duties it is to obtain from the registrar a list of all the births registered, to visit the mothers, and leave instructions on the feeding of infants. The high rate of infant mortality in England and Wales (153 per 1,000) has long been a blot upon our sanitary system, and the efforts made by the St. Helen's authorities to secure a better condition of things will be keenly watched from all parts of the kingdom.

### Plague in Glasgow.

The reported occurrence of a group of cases of plague at Glasgow is a matter which must give rise to considerable anxiety and uneasiness, bearing out, as it does, what we have several times said in regard to the subtle character of the infection of this disease, and the extreme difficulty, on the one hand, of preventing its entry into any country by inspection at the ports, and, on the other, of controlling its dissemination by any of the ordinary methods of notification and isolation. Nothing could have been more natural than that, with the free intercommunication which exists between British ports and places where plague has of late years been so prevalent, cases should now and again occur on board ship, or even that persons suffering from the disease should be landed on our shores. The wonder is that this has occurred so seldom as has been the case. Nor has there seemed any special reason for alarm when it has occurred. The cases have been promptly isolated and there has been the end of the matter. In Glasgow, however, the affair, as reported, seems to stand on a different footing. It appears that a week or ten days ago a girl died of what was then thought to be pneumonia. The following day her mother and two brothers also became ill, and were removed to the Belvedere Hospital, where symptoms of plague were found to exist—the diagnosis being afterwards confirmed by Professor Muir—and where one of the brothers died. Tracing the history of these cases back it seems that a few days before the girl became ill the mother had attended a wake in the house of a dock labourer, and that seems as far back as the report goes at present. If, then, the statement that these patients have really been suffering from plague should be confirmed, and we have no reason to doubt it, what we have to deal with is not an imported case or group of cases which can be isolated, and the infection of which can be at once destroyed, but a disease which has already got a footing, and, as usual, in an old building in a poor quarter of the town. The importance of an outbreak of plague must be measured not by the virulence of the cases nor by their number, but by the extent to which the evidence points to the disease having already been in existence in a more or less latent fashion for a time long enough to enable the rats and other parasites of man to have become infected. It is the fact that plague is not an exclusively human disease that makes it so difficult to control it by measures which apply to man alone.

### Our Dealings with the Dead.

One of the problems of town life which most urgently calls for solution is how to deal with the bodies of the dead. A report has recently been made by Dr. Young, assistant medical officer to the London County Council, on the sanitary condition of cemeteries and burial grounds, which shows how very defective the existing arrangements are, and how necessary it is that some improvement should be introduced without delay. It is a disagreeable fact that the dead far outnumber the living, and that by yielding to the prejudice so many people feel in favour of preserving the bodies of their relatives, every great city of the living is building up a still greater city of the dead, rotting and festering around it. All this is the result of that delightful thing we call civilisation. Nature if let alone knows well enough how to deal with its dead. A body just simply buried in a few inches of earth gradually and peacefully disintegrates, and is before long resolved into its elements, so that nothing but perhaps a few crumbling bones is left to indicate the place of burial. Religious sentiment, however, custom and tradition, and often, it

**ANNOTATIONS**—*continued.*

is to be feared, mere vulgar aping of the great, has led to a method of disposing of the dead which is so offensive that we doubt whether even fashion would induce people to submit the bodies of their loved ones to it if they for a moment recognised the disgusting nature of the whole performance. Unfortunately, as things stand at present, proper and reasonable methods of burial are actually prohibited by law, the regulations requiring that no corpse shall be buried at less than four feet from the surface. Downwards, however, there seems to be no limit, and thus it happens that "common" graves are dug for twenty feet deep or so, and that the bodies of men and women, friends and strangers, just as they happen to come, are piled in one on top of the other, and are left to putrefy in each other's juices. Not only is all this very horrid, but it is all quite unnecessary. Mr. Seymour Haden has long ago shown how rapidly the whole of the body, except some of the bones, is destroyed and turned to innocuous mould when buried near the surface of the ground, and buried so that the soil can come in contact with it; and a careful perusal of Dr. Young's report leaves us convinced that, in seeking for a proper and orderly method of disposing of the dead, our choice must lie between cremation and earth to earth burial, as superficial burial in readily decomposable coffins is called. So far as sentiment is concerned, one would think that anything would be better than the lead and oak coffins, the bricked graves, the common interment, the deeply-dug pits, the rotting bodies, which are described in this report and seem to be inherent in the present system; while from the sanitary point of view it is obvious that something must be done before long to prevent our towns becoming walled in by a ring of ever-growing cemeteries.

### The Workmen's Compensation Act.

It would appear that few things are more difficult than to draft an Act of Parliament in such a manner as to carry out the objects aimed at without producing new evils, greater perhaps than those whose abolition was intended. The Workmen's Compensation Act of 1897 was undoubtedly framed with good intentions, and it was framed with care. It even went so far as to provide for the employment of medical referees to aid in arriving at a true solution of some of the complex medical problems which were sure to arise. Judges, however, are not very fond of referees or assessors. They like "evidence," and on the strength of the evidence offered in certain cases some very interesting decisions have been given which vastly widen the scope of the Act, tending, as they do, to show that, however much a man's disablement may be due to disease rather than to injury, the employer must pay if the "accident" was responsible for rousing the disease into activity. The leading case was, we believe, that of a gouty man in whom an accident was followed by a chronic gouty condition by which he was disabled. The outcome of this interpretation of the law has been somewhat serious, and will probably, as years go on, become more serious still. It is becoming apparent how grave is the risk to employers of having ailing or feeble men about their works; and the hardship long felt by elderly workmen, who find themselves ousted by younger competitors in consequence of unbending trades union rules, is being accentuated by the operation of this Act, which almost forces an employer to weed his workers. A young man traps his finger and is well in a fortnight, thus not coming under the Act at all. Another man who happens, perhaps, to have some constitutional disease, meets with exactly the same accident, but, instead of getting well in a fortnight, he never again is able to do quite the work he did before, and is therefore given an annuity for life at the cost of his employer. Need we wonder, then, that the employer, or the insurance society which by the rates it offers influences the employer, takes good care to weed out as quickly as possible all those who show signs of age or weakness? The Act is good for those who get the pension, but bad for the rest, for it tends to shorten the average length of wage-earning life, and to hasten the time when the worker becomes dependent upon others.

### Responsibility for Infection.

A case was tried a little time ago the judgment in regard to which seems to a common man, although, probably, not to a lawyer, to bear a somewhat wide application. It was an action against the vestry of Camberwell for negligence in allowing sewage to escape from one of their sewers into the plaintiff's cellars, and the outcome of it was that the vestry had to pay damages to the tune of £100. The vestry did not do this evil deed, but they allowed it to happen; in other words, it was by their default that it occurred. Now, the common man, knowing that Parliament has given to the sanitary authorities powers to prevent people exposing themselves in public places when suffering from infectious disease, not unnaturally concludes that if a sanitary authority is liable for the damage resulting from default in looking after its drains, far more should it be liable for the consequence of its remissness in not putting the law in force in regard to infection, and not protecting its citizens from being infected while walking in its streets. We doubt whether the point has been raised, but it at least seems open to argument. If a respectable law-abiding citizen when scarlet fever breaks out in his family notifies the disease, keeps away from his work, sacrifices his wages, keeps his children from school much to his own discomfort, and shuts them up in the house greatly to the injury of their health, and if he does all this, not for his own profit, but because the law declares that he shall do so for the protection of his neighbours and the town at large, it is rather hard that as soon as they go out they should be exposed to the chance of catching, say, diphtheria because, from the laxity with which the law is administered, his neighbour's children are scattering infection broadcast in the streets. Undoubtedly the sanitary authority have the power to prosecute when people fail to notify, or when they expose themselves while infectious in a public place, and many a law-abiding citizen would like to feel that he also had a remedy should he catch an infectious disease in consequence of the neglect of the authority to put the law in force. We fear, however, the case would be hard to prove.

